# Physical Location of New PCR-Based Markers and Powdery Mildew Resistance Gene(s) on Rye (*Secale cereale* L.) Chromosome 4 Using 4R Dissection Lines

**DOI:** 10.3389/fpls.2017.01716

**Published:** 2017-10-10

**Authors:** Qiong Duan, Yang Yang Wang, Ling Qiu, Tian Heng Ren, Zhi Li, Shu Lan Fu, Zong Xiang Tang

**Affiliations:** ^1^Province Key Laboratory of Plant Breeding and Genetics, Sichuan Agricultural University, Chengdu, China; ^2^Institute of Ecological Agriculture, Sichuan Agricultural University, Chengdu, China; ^3^College of Life Sciences, Sichuan Agricultural University, Ya’an, China

**Keywords:** wheat, rye, 4R dissection line, PCR-based markers, physical map, powdery mildew

## Abstract

Rye (*Secale cereale* L.) 4R chromosome contains elite genes that are applicable for wheat (*Triticum aestivum* L.) cultivar improvement. PCR-based 4R-specific markers can benefit the detection of elite genes on 4R in wheat backgrounds. In this study, a new fluorescence *in situ* hybridization (FISH) map of the 4R^Ku^ chromosome of rye Kustro has been constructed. A set of 4R^Ku^ dissection lines was obtained and 301 new 4R^Ku^-specific markers were developed using specific length amplified fragment sequencing (SLAF-seq) technology. These markers were combined with the 99 4R^Ku^-specific markers previously developed, and were physically mapped to 4R^Ku^ chromosome using the new FISH map and the 4R^Ku^ dissection lines. A total of 338 of the 400 markers have been successfully mapped to six regions of 4R^Ku^ chromosome. Additionally, the powdery mildew resistance gene(s) on the 4RL^Ku^ arm was located to the segment between L.4 and L.8, the same region where 115 4RL^Ku^-specific markers were mapped. The markers developed in this study can be used to identify a specific segment of 4R chromatin in wheat backgrounds, help construct a high-density physical map of 4R chromosome, and facilitate the utilization of elite genes on 4R chromosome in wheat breeding programs.

## Introduction

Rye (*Secale cereale* L.) is an important gene source for wheat (*Triticum aestivum* L.) cultivar improvement. Only the short arm of rye chromosome 1R has been widely used to develop wheat cultivars through 1BL.1RS or 1AL.1RS translocation chromosomes (Berzonsky et al., 1991; Rabinovich, 1998; Kumar et al., 2003; Landjeva et al., 2006). In fact, other rye chromosomes also contain elite genes that can be used for wheat improvement. For example, 4R chromosome contains disease- and insect-resistance genes. Lukaszewski et al. (2001) reported that the 4RL arm of *Secale montanum* Guss carried a Russian wheat aphid (RWA) resistance gene. New powdery mildew resistance gene(s), which differ from previously reported rye genes, were located on 4R chromosome of rye cultivar German White (An et al., 2013). It has been reported that the 4RL arm of rye Kustro also possesses powdery mildew resistance gene(s) (Fu et al., 2014). Additionally, the 4R chromosome of rye Imperial carries at least two genetic factors that have positive effects on wheat pollination traits (Nguyen et al., 2015). The addition of ‘Kriszta’ chromosome 4R to wheat genome can increase the total protein content (Schneider et al., 2016). However, the utilization of elite genes on 4R chromosome for wheat improvement is difficult because of compensation issues and the very low recombination frequency of the 4R chromosome with its wheat homoeologues (Lukaszewski et al., 2001). Because of these issues, the only available approaches are constructing wheat-rye small-segment translocation lines and cloning rye elite genes. Elite genes can be utilized efficiently when rye chromosomal segments have been transferred into wheat and precisely identified. A well-saturated molecular linkage map can be used for gene tagging. Rye chromosome-specific markers are beneficial to the effective application of rye elite genes in wheat breeding programs, however, very few 4R-specific markers, especially PCR-based and agarose gel electrophoresis-based markers, have been developed so far.

In this study, New PCR-based and 4R^Ku^-specific markers using specific length amplified fragment sequencing (SLAF-seq) technology were developed. Subsequently, these new markers were physically mapped onto six regions (bins) on 4R^Ku^ using 4R^Ku^ dissection lines in a wheat background.

## Materials and Methods

### Plant Materials

The octoploid triticale line MK was developed by crossing common wheat *T. aestivum* L. Mianyang 11 (MY11) with rye *S. cereale* L. Kustro. Progeny were produced by controlled backcrossing of MK with MY11, followed by self-fertilization. From these progeny, seven wheat-rye monosomic addition lines (MA1R^Ku^-MA7R^Ku^ lines), a 4RS^Ku^ monotelosomic addition line (MTA4RS^Ku^) and a 4RL^Ku^ monotelosomic addition line (MTA4RL^Ku^) were detected (Li et al., 2016; Qiu et al., 2016). Some of the MK seeds were irradiated with fast neutrons at the Institute of Nuclear Physics and Chemistry, China Academy of Engineering Physics, Mianyang, China. The irradiated MK seeds were used as recipients to cross with common wheat *T. aestivum* L. Chuannong 27 (CN27), and line 12FT2115 was selected from the progeny of this cross combination (Fu et al., 2014). Some of the selfed progeny (seeds) of lines 12FT2115 and MTA4RL^Ku^ were irradiated with ^60^Co-γ rays at the Biotechnology and Nuclear Technology Research Institute, Sichuan Academy of Agricultural Sciences, China. Common wheat *T. aestivum* L. Chinese Spring (CS) was used as a control.

### Cytological Techniques and *In Situ* Hybridization

Non-denaturing fluorescence *in situ* hybridization (ND-FISH) technology was used to analyze root-tip metaphase cells. Oligonucleotide probes containing Oligo-1162, Oligo-pSc200, Oligo-pSc250, Oligo-pSc119.2-1, and Oligo-pTa535-1 were synthesized following the methods described by Tang et al. (2014) and Fu et al. (2015). ND-FISH was carried out following the procedure described by Fu et al. (2015). Probes Oligo-1162, Oligo-pSc200, Oligo-pSc250 and Oligo-pTa535-1 were 5′-end-labeled with 6-carboxytetramethylrhodamine (Tamra). Probe Oligo-pSc119.2-1 was 5′-end-labeled with 6-carboxyfluorescein (6-FAM). Additionally, a synthetic oligonucleotide probe (AAC)_6_ was used and was 5′-end-labeled with Cy5. Metaphase chromosomes of the root-tips were prepared following the methods described by Han et al. (2006). Images were made using an epifluorescence Olympus BX51 microscope, which was equipped with a cooled charge-coupled device camera and with the HCIMAGE Live software (version 2.0.1.5). Images were processed using Adobe Photoshop CS 3.0.

### Development of PCR-Based Markers

Genomic DNAs of *S. cereale* L. Kustro and MA4R^Ku^ were sequenced using the SLAF-seq technique (Biomarker, Beijing, China). The sequencing procedure followed the methods described by Chen et al. (2013), with some modifications. Genomic DNAs of Kustro and MA4R^Ku^ were digested using the restriction enzyme, *Hae*III. Subsequently, a Quick Spin column (Qiagen) was used to purify the samples and then run out on a 2% agarose gel. Fragments between 450 to 500 bp were isolated using a Gel Extraction Kit (Qiagen). These isolates were used in a PCR reaction described by Chen et al. (2013). Amplicons with the sizes between 450 to 500 bp were excised and diluted for sequencing, and they were identified, filtered, clustered and corrected following the methods described by Chen et al. (2013). The pair-end reads derived from Kustro and MA4R^Ku^ were compared with wheat A genome, D genome and *T. aestivum* L. Chinese Spring (supported by Biomarker, Beijing, China) sequences using SOAP software (Li et al., 2009). The pair-end reads with low wheat homology were kept. Finally, after comparing specific pair-end reads of Kustro and MA4R^Ku^, the 4R^Ku^ specific pair-end reads were obtained. Primers were designed according to the 4R^Ku^ specific pair-end reads using the software Primer 3 (version 4.0). For primers, optimal melting temperature and size values were set to 60°C and 20 bases, respectively.

### Physical Location of 4R-Specific Markers

Markers, whose products presented in Kustro and MA4R^Ku^, but were absent in CS, MY11, CN27, MA1R^Ku^-MA3R^Ku^ and MA5R^Ku^-MA7R^Ku^, were regarded as 4R^Ku^-specific markers. These markers were located to specific regions of the 4R^Ku^ chromosome using the 4R^Ku^ dissection lines. Additionally, 99 4R^Ku^-specific markers that were previously developed by Qiu et al. (2016) were located on specific regions of the 4R^Ku^ chromosome using the 4R^Ku^ dissection lines.

### PCR Analysis

The PCR amplifications were carried out according to the procedure described by Li et al. (2016). The amplicons were electrophoresed on 2% agarose gels in 1 × TAE buffer. For each of the primer pairs used in this study, PCR reactions were repeated three times.

### Powdery Mildew Resistance Test

The resistance of 4R^Ku^ dissection lines and parental wheat MY11 and CN27 to powdery mildew was evaluated. Plants were grown in two growing seasons (2015-2016 and 2016-2017) in Qionglai, Sichuan, China. The materials were naturally infected by powdery mildew, and infection types (IT) were scored according to the standard described by Fu et al. (2014).

## Results

### FISH Map of Chromosome 4R

**FIGURE 1:**
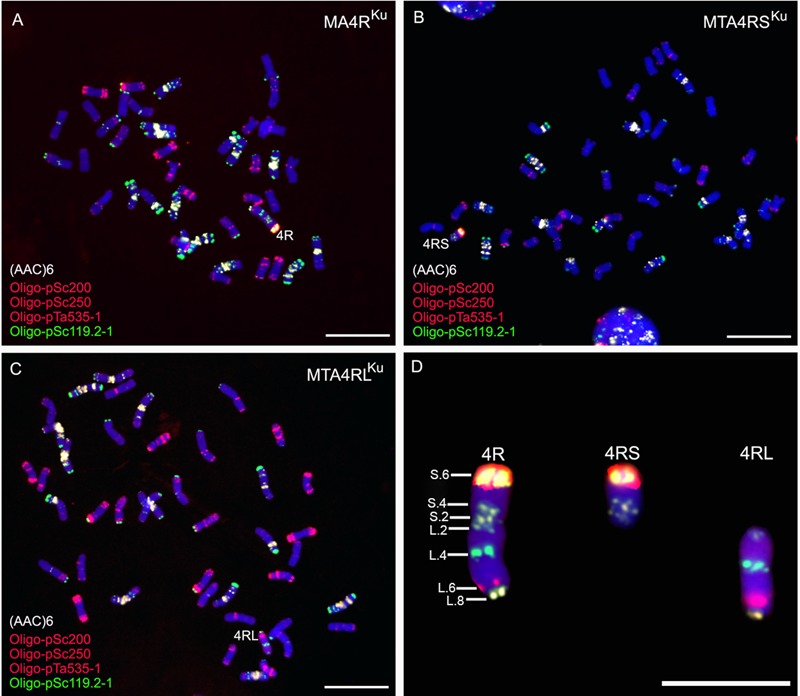
ND-FISH analysis using probes Oligo-pSc119.2-1 (green), Oligo-pTa535-2 (red), Oligo-pSc200 (red), Oligo-pSc250 (red) and (AAC)_6_ (white) to identify MA4R^Ku^, MTA4RS^Ku^ and MTA4RL^Ku^. **(A)** Line MA4R^Ku^ contains 42 wheat chromosomes and a 4R chromosome. **(B)** Line MTA4RS^Ku^ contains 42 wheat chromosomes and a 4RS arm. **(C)** Line MTA4RL^Ku^ contains 42 wheat chromosomes and a 4RL arm. **(D)** Cut-pasted 4R^Ku^ chromosome, 4RS^Ku^ arm and 4RL^Ku^ arm. A new FISH map was constructed according to Oligo-pSc119.2-1, Oligo-pTa535-2, Oligo-pSc200, Oligo-pSc250 and (AAC)_6_ signals. Chromosomes were counterstained with DAPI (blue). Scale bar is 10 μm.

Repetitive DNA sequence (AAC)_6_, combined with three oligonucleotides Oligo-pSc119.2-1, Oligo-pSc200 and Oligo-pSc250, were used as probes to analyze the root tip metaphase chromosomes of lines MA4R^Ku^, MTA4RS^Ku^, and MTA4RL^Ku^. The signals of probes Oligo-pSc119.2-1, Oligo-pSc200 and Oligo-pSc250 on the telomere of 4RS^Ku^ were very strong. Oligo-pSc119.2-1 produced a strong signal at the interstitial site of 4RL^Ku^, and Oligo-pSc200 and Oligo-pSc250 produced clear signals at the sub-telomeric region of 4RL^Ku^ (**Figure 1**). Probe (AAC)_6_ had two signal sites on 4RS^Ku^, one signal site on the pericentromeric region of 4RL^Ku^ and one signal site on the telomere of 4RL^Ku^ (**Figure 1**). Based on an *in situ* hybridization map of 4R chromosome constructed by Cuadrado et al. (1995), the signal sites of probes (AAC)_6_, Oligo-pSc119.2-1, Oligo-pSc200 and Oligo-pSc250 were numbered, and a new FISH map of 4R chromosome was constructed (**Figure 1D**).

### Isolation of 4R^Ku^ Dissection Lines

**FIGURE 2:**
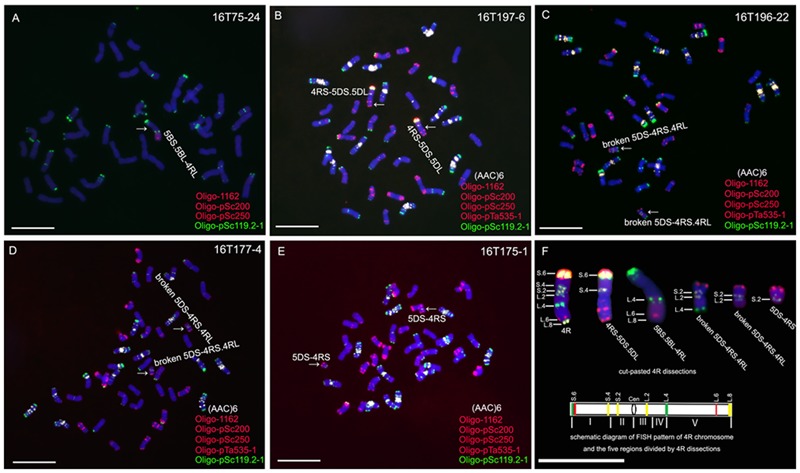
ND-FISH analysis using probes Oligo-pSc119.2-1 (green), Oligo-pTa535-2 (red), Oligo-pSc200 (red), Oligo-pSc250 (red) and (AAC)_6_ (white) to identify 4R^Ku^ dissection lines. **(A)** 5BS.5BL-4RL translocation line 16T75-24. **(B)** 4RS-5DS.5DL translocation line 16T197-6. **(C)** Line 16T196-22 contains broken 5DS-4RS.4RL chromosomes. **(D)** Line 16T177-4 contains broken 5DS-4RS.4RL chromosomes. **(E)** Line 16T175-1 contains 5DS.4RS small translocation chromosomes. **(F)** Cut-pasted 4R^Ku^ dissections and the schematic diagram of FISH pattern of the 4R^Ku^ chromosome. The FISH signals are numbered. In the schematic diagram, five regions of the 4R^Ku^ chromosome were indicated by Roman numerals, green bands represent Oligo-pSc119.2-1 signals, red bands represent Oligo-pSc200 and Oligo-pSc250 signals, yellow bands represent (AAC)_6_ signals, and ‘Cen’ represents centromere. Chromosomes were counterstained with DAPI (blue). Scale bar is 10 μm.

Line 16T75-24, containing a 5BS.5BL-4RL translocation chromosome, was detected from the irradiated seeds of line MTA4RL^Ku^ (**Figure 2A**). A homozygous 4RS-5DS.5DL translocation line, 16T197-6, was obtained from the selfed progeny of irradiated 12FT2115 (**Figure 2B**), and three kinds of broken 5DS-4RS.4RL chromosomes were detected in lines 16T196-22, 16T177-4 and 16T175-1, respectively (**Figures 2C–E**). On the 5BS.5BL-4RL translocation chromosome, the breakpoint of 4RL^Ku^ was located at the Oligo-pSc119.2 signal site and the segment from L.4 to L.8 was retained (**Figures 2A,F**). On the 4RS-5DS.5DL chromosomes, the segments from S.4 to S.6 of the 4R^Ku^ chromosome were kept (**Figures 2B,F**). Breakpoints on the pair of 5DS-4RS.4RL chromosomes in line 16T196-22 were located at the Oligo-pSc119.2 signal sites of the 4RL^Ku^, and the segments between L.4 and L.8 were lost as were segments between S.4 and S.6 of the short arm (**Figures 2C,F**). The breakpoints on the pair of 5DS-4RS.4RL chromosomes in line 16T177-4 were located on the regions between L.2 and L.4, and the segments between the breakpoints and L.8 were absent as were segments of the short arm from S.4 to S.6 (**Figures 2D,F**). In line 16T175-1, the 5DS-4RS.4RL chromosomes were broken at the centromeres, and this line contained two 5DS-4RS small translocation chromosomes (**Figures 2E,F**). Therefore, the wheat–rye translocation chromosomes in lines 16T75-24, 16T197-6, 16T196-22, 16T177-4 and 16T175-1 composed a set of 4R^Ku^ dissection lines, and the 4R^Ku^ chromosome was divided into five regions (**Figure 2F**).

### Development of 4R-Specific Markers

**FIGURE 3:**
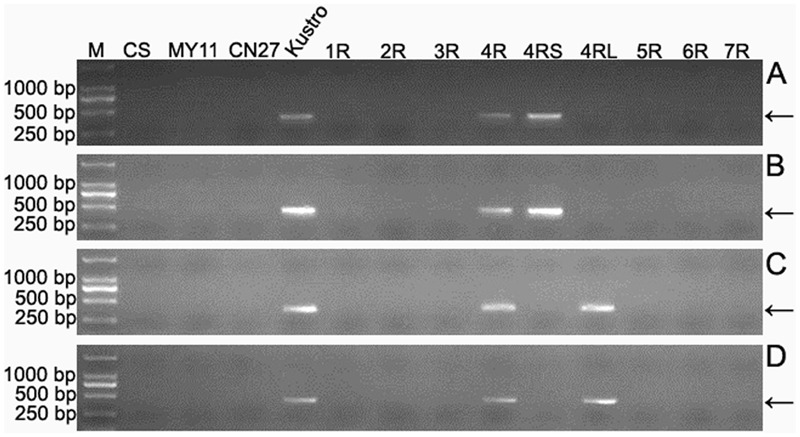
Identification of 4R^Ku^-specific, 4RS^Ku^-specific and 4RL^Ku^-specific markers using CS, MY11, CN27, Kustro, MTA4RS^Ku^, MTA4RL^Ku^ and MA1R^Ku^–7R^Ku^. **(A)** Products amplified by primer pair KU-4R.429. **(B)** Products amplified by primer pair KU-4R.732. **(C)** Products amplified by primer pair KU-4R.012. **(D)** Products amplified by primer pair KU.170. Primer pairs KU-4R.429 and KU-4R.732 represent the 4RS^Ku^-specific markers. Primer pairs KU-4R.012 and KU.170 represent the 4RL^Ku^-specific markers. M: DNA marker. CS: Chinese Spring. MY11: Mianyang 11. CN27: Chuannong 27. Kustro: rye kustro. 1R-7R: MA1R^Ku^–7R^Ku^. 4RS: MTA4RS^Ku^. 4RL: MTA4RL^Ku^. Arrows indicate the target bands.

Seven wheat–rye monosomic addition lines including MA1R^Ku^, MA2R^Ku^, MA3R^Ku^, MA4R^Ku^, MA5R^Ku^, MA6R^Ku^, and MA7R^Ku^ were used to identify 4R^Ku^-specific markers. From the 33,577 4R^Ku^-specific pair-end reads, 780 reads were randomly selected for designing primers. Three hundred and one of the 780 primer pairs amplified specific bands from Kustro and MA4R^Ku^, but not from CS, MY11, CN27, MA1R^Ku^-MA3R^Ku^ and MA5R^Ku^-MA7R^Ku^ (**Figure 3**). The 301 primer pairs were regarded as 4R^Ku^-specific markers (Supplementary Table S1). Using lines MTA4RS^Ku^ and MTA4RL^Ku^, 128 and 173 of the 301 markers were located on the 4RS^Ku^ and 4RL^Ku^ arms, respectively (**Figure 3** and Supplementary Table S2).

### Physical Mapping of 4R^Ku^-Specific Markers

**FIGURE 4:**
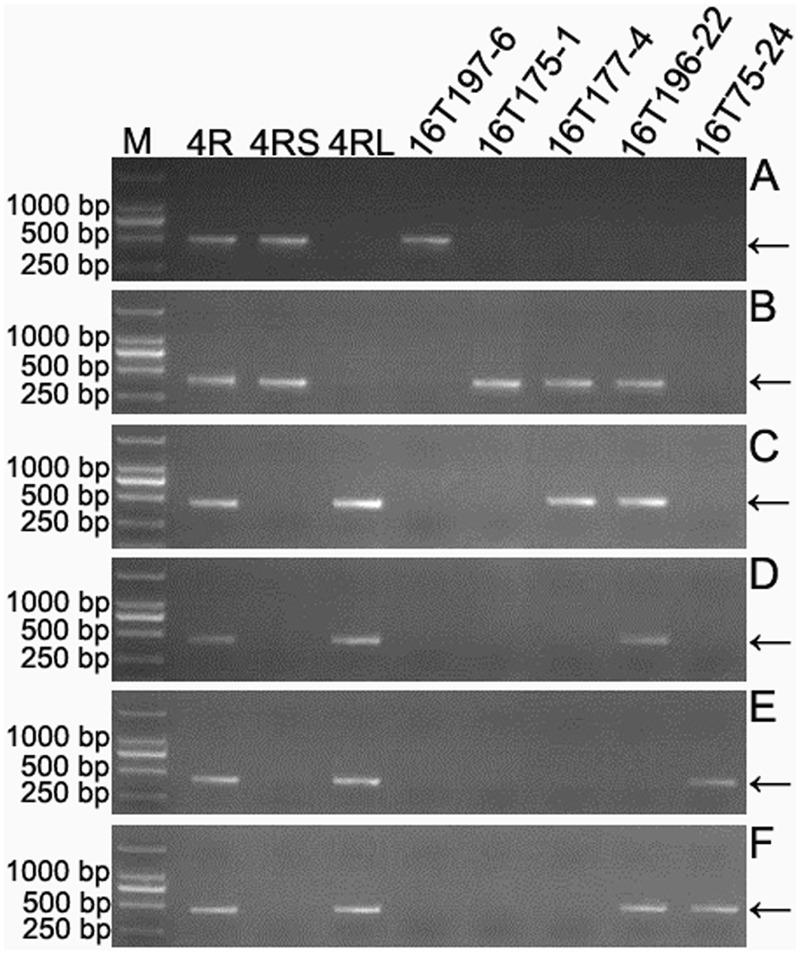
Physically localized 4R^Ku^-specific markers using 4R^Ku^ dissection lines. **(A)** Products amplified by primer pair KU.737. **(B)** Products amplified by primer pair KU-4R.115. **(C)** Products amplified by primer pair KU-4R.149. **(D)** Products amplified by primer pair KU-4R.563. **(E)** Products amplified by primer pair KU-4R.004. **(F)** Products amplified by primer pair KU.1058. Primer pairs KU.737, KU-4R.115, KU-4R.149, KU-4R.563, KU-4R.004 and KU.1058 respectively represent the markers that mapped to the six regions including I, II, III, IV, V, and the region cross IV and V. M: DNA marker. 4R: MA4R^Ku^. 4RS: MTA4RS^Ku^. 4RL: MTA4RL^Ku^. Arrows indicate the target bands.

**FIGURE 5:**
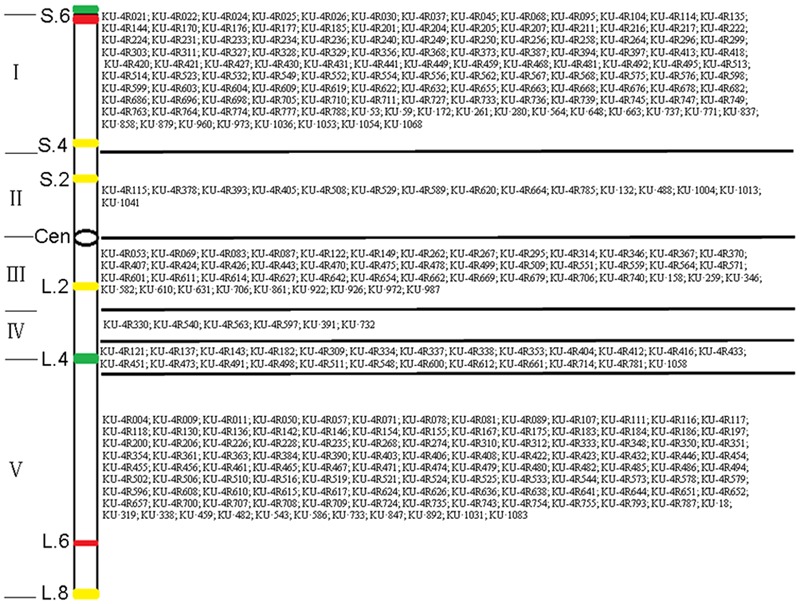
Physical map of 338 4R^Ku^-specific markers in six regions on 4R^Ku^ chromosome based on PCR amplification using 4R^Ku^ dissection lines. The six regions are divided by the five dark lines. In each region, the names of the 4R^Ku^-specific markers are listed on the right. The values on the left indicate numbered sites, which were determined by the FISH signals. The Roman numerals on the left indicates the five regions of the 4R^Ku^ chromosome divided by FISH signals. In the schematic diagram, green bands represent Oligo-pSc119.2-1 signals, red bands represent Oligo-pSc200 and Oligo-pSc250 signals, yellow bands represent (AAC)_6_ signals, and ‘Cen’ represents centromere.

The 301 4R^Ku^-specific markers obtained in this study were physically located on six regions of the 4R^Ku^ chromosome using lines 16T75-24, 16T175-1, 16T177-4, 16T196-22 and 16T197-6 (**Figure 4** and Supplementary Table S2). Additionally, the 31 4RS^Ku^-specific and 68 4RL^Ku^-specific markers developed by Qiu et al. (2016) were also mapped to the six regions of the 4R^Ku^ chromosome (**Figure 4** and Supplementary Table S2). Among the 159 (128 + 31) 4RS^Ku^-specific markers, 128 amplified 4RS^Ku^-specific bands from line 16T197-6, but not the other four lines, and 15 amplified 4RS^Ku^-specific bands from lines 16T175-1, 16T177-4, and 16T196-22, but not lines 16T197-6 and 16T75-24 (**Figures 4A,B**). Therefore, the 128 markers were mapped to region I and the 15 markers were mapped to region II (**Figure 5**). There were 16 4RS^Ku^-specific markers that could not amplify their products from any of the five dissection lines, therefore, they could not be mapped to any region of the 4RS^Ku^ arm (Supplementary Table S2). For the 241 (173 + 68) 4RL^Ku^-specific markers, 49 markers only amplified their special products from line 16T77-4, carrying the proximal long arm segment from the centromere to L.2, and line 16T196-22, carrying the proximal long arm segment from the centromere to L.4 (**Figure 4C** and Supplementary Table S2), six markers amplified 4RL^Ku^-specific bands from line 16T196-22 (**Figure 4D** and Supplementary Table S2), 115 markers only amplified their specific bands from line 16T75-24, which carries the long arm distal segment from L.4 to the telomere (**Figure 4E** and Supplementary Table S2), and 25 markers amplified 4RL^Ku^-specific bands from both the lines 16T75-24 and 16T196-22 but not the lines 16T175-1, 16T177-4 and 16T197-6 (**Figure 4F** and Supplementary Table S2). Therefore, the 49, six and 115 markers were mapped to regions III, IV, and V, respectively (**Figure 5**). The 25 markers were located near the site L.4 and across the IV and V regions (**Figure 5**). The remaining 46 4RL^Ku^-specific markers did not amplify their specific band from any of the five deletion lines and they could not be mapped to any region of the 4RL^Ku^ arm (Supplementary Table S2).

### Physical Location of the Powdery Mildew Resistance Gene(s) on 4RL

**FIGURE 6:**
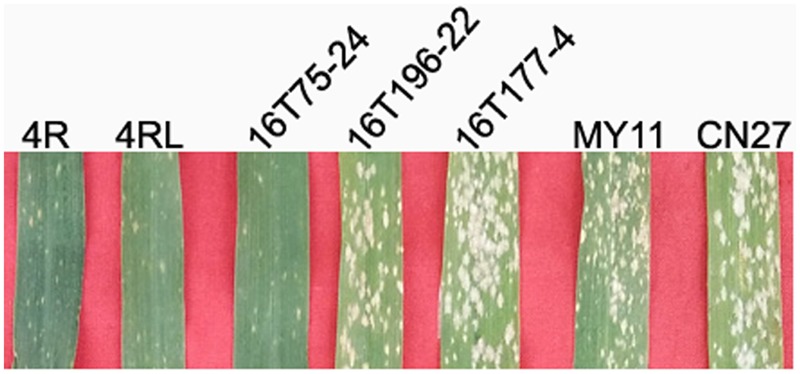
Powdery mildew resistance testing. MY11, CN27, line 16T177-4 and line 16T196-22 are highly susceptible to powdery mildew. Lines MA4R^Ku^, MTA4RL^Ku^, 16T75-24 are highly resistant to powdery mildew.

The resistance of the lines MA4R^Ku^, MTA4RL^Ku^, 16T75-24, 16T177-4, 16T196-22, and parental wheat MY11 and CN27 to powdery mildew was tested in the field. Results showed that MA4R^Ku^, MTA4RL^Ku^ and 16T75-24 were highly resistant to powdery mildew (IT = 1), and 16T177-4, 16T196-22, MY11 and CN27 were highly susceptible (IT = 4) (**Figure 6**). Therefore, the powdery mildew resistance gene(s) on 4RL^Ku^ was localized to the region V (segment between L.4 and L.8).

## Discussion

### 4R-Specific Markers

Isozyme, protein, RFLP, DArT, and SSR markers have been used to develop genetic maps of 4R chromosome (Benito et al., 1994; Korzun et al., 1998; Saal and Wricke, 1999; Korzun et al., 2001; Ma et al., 2001; Milczarski et al., 2007; Gustafson et al., 2009; Milczarski et al., 2011; Milczarski et al., 2016). However, rare specific markers can be used to detect 4R chromosome in wheat backgrounds. Three *Secale cereale* inter-microsatellite (SCIM) markers were located on 4R chromosomes (Camacho et al., 2005). A wheat simple sequence repeat (SSR) marker, X*gwm*260, can produce a 4R-specific band (Fu et al., 2010). Two 4R-specific markers were developed using *Eco*O109I primers (Tomita and Seno, 2012). Six 4R-specific markers derived from expressed sequence tags (ESTs) were obtained (Xu et al., 2012). Li et al. (2013) developed eight 4R-specific markers using the polymerase chain reaction (PCR)-based landmark unique gene (PLUG) system. Qiu et al. (2016) developed 101 4R^Ku^-specific markers using SLAF-seq technology. Almost all of the 4R-specific markers mentioned above are PCR-based markers, and are easier to perform than RFLP, AFLP, and DArT markers. Additionally, these markers not only can be used to distinguish 4R chromatin in wheat backgrounds, but also can be used to construct a map of 4R chromosome. Lukaszewski et al. (2001) attempted to map the genetic position of the RWA resistance locus on 4RL using 4RL arms, which were derived from two different rye lines. However, this attempt was unsuccessful, partially because of the absence of cytologically identifiable recombinants (Lukaszewski et al., 2001). This case indicates the current lack of and the immediate need for additional 4R-specific markers. In this study, 301 new PCR-based and 4R^Ku^-specific markers were developed, adding to the specific markers for distinguishing 4R chromatin in wheat backgrounds and 4R chromosome mapping.

### Physical Location of 4R^Ku^-Specific Markers Using Dissection Lines

It has been reported that genetic maps based on recombination rates can not represent the actual physical location of genes and molecular markers on chromosomes (DeScenzo and Wise, 1996). Physical maps complement genetic maps. For wheat and its relatives, chromosomal dissection or deletion lines are useful for determining the physical location of genes and molecular markers (Tsuchida et al., 2008). An array of chromosome deletion stocks have been used to construct physical maps of common wheat (Endo and Gill, 1996). Many molecular markers were physically located on specific segments of barley chromosomes (Ashida et al., 2007; Sakata et al., 2010; Ishihara et al., 2014). Pu et al. (2015) obtained a series of structural aberrations of *Thinopyrum bessarabicum* chromosome 4J and used them to physically map 101 4J-specific markers and the blue-grained gene *Bathb*. *Agropyron cristatum* chromosome 6P-specific STS markers were physically located in 14 regions of this chromosome using 6P deletion lines (Song et al., 2016). Rye chromosome dissection lines were also used to physically map rye-specific markers that are mainly restricted to chromosomes 1R, 2R, and 6R (Kofler et al., 2008; Tsuchida et al., 2008; Gyawali et al., 2009, 2010; Zhuang et al., 2015; Li et al., 2016). In this study, a new FISH map of the 4R^Ku^ chromosome was constructed using probes (AAC)_6_, Oligo-pSc119.2-1, Oligo-pSc200 and Oligo-pSc250. The 4R^Ku^ dissection lines combined with the new FISH map of the 4R^Ku^ chromosome were used to physically map 400 4R^Ku^-specific markers to five regions (**Figure 5**). Physically locating these markers is beneficial for selecting introgressed 4R small segments in wheat backgrounds to create translocation lines.

### Variation of 4R^Ku^-Specific Markers

During the development of rye-specific markers, some rye chromosome-specific markers that were identified using wheat–rye addition lines were often not able to be located to rye chromosome arms or given chromosome segments. For example, some 6R-specific bands could not be amplified from either 6RS or 6RL telosomic addition lines (Xu et al., 2012). Two of the 101 4R^Ku^-specific markers could not be physically mapped to 4RS^Ku^ and 4RL^Ku^ arms (Qiu et al., 2016). It is possible that this was caused by variations in the structures of rye chromosome arms during the procedure of development of wheat–rye telosomic addition lines (Qiu et al., 2016). In this study, there were 16 4RS^Ku^-specific and 46 4RL^Ku^-specific markers could not be mapped to any regions of 4R^Ku^ chromosome using the 4R^Ku^ dissection lines. This indicated that the 4R^Ku^ chromosome segments in these dissection lines were altered during irradiation.

### Localization of Powdery Mildew Resistance Gene(s) on 4RL

It has already been reported that 4R chromosome of rye cultivar German White carried powdery mildew resistance gene (s) (An et al., 2013). Subsequently, it was reported that 4R chromosome of rye Kustro also possesses powdery mildew resistance gene(s), which was mapped on the long arm of 4R^Ku^ (4RL^Ku^) (Fu et al., 2014). In this study, the powdery mildew resistance gene(s) on 4RL of Kustro was physically mapped to the V region (segment between L.4 and L.8). Therefore, the powdery mildew resistance gene(s) on 4RL^Ku^ has been located to a more explicit segment on 4RL^Ku^ arm, and 115 PCR-based and 4RL^Ku^-specific markers that were located to this region has also been developed. These 115 markers will be helpful for localizing the powdery mildew resistance gene(s) on 4RL^Ku^ arm in wheat breeding programs. However, more 4R dissection lines are needed to localize the powdery mildew resistance gene(s) to a smaller region on 4RL arm.

## Conclusion

A new FISH map of 4R^Ku^ chromosome has been constructed. A set of 4R^Ku^ dissection lines was obtained and 301 new 4R^Ku^-specific markers were developed. The 301 markers were combined with the 99 4R^Ku^-specific markers developed previously, and were physically mapped to 4R^Ku^ chromosome using the new FISH map of 4R^Ku^ chromosome and the 4R^Ku^ dissection lines. 338 of the 400 markers have been successfully mapped to six regions of 4R^Ku^ chromosome. In addition, the powdery mildew resistance gene(s) on 4RL^Ku^ arm has been located to a clearly defined region and 115 markers will be helpful for the further localizing the powdery mildew resistance gene(s). The markers developed in this study have enriched the collection of markers that can specifically identify the 4R chromatin in wheat backgrounds and can be used to construct high-density map of 4R chromosome.

## Author Contributions

SF and ZT designed the study, analyzed the data and wrote the manuscript. QD, YW, and LQ designed the primers and performed experiments. TR and ZL performed experiments.

## Conflict of Interest Statement

The authors declare that the research was conducted in the absence of any commercial or financial relationships that could be construed as a potential conflict of interest.

## References

[B1] An D. G. Zheng Q. Zhou Y. L. Ma P. T. Lv Z. L. Li L. H. (2013). Molecular cytogenetic characterization of a new wheat-rye 4R chromosome translocation line resistant to powdery mildew. *Chromosome Res.* 21 419–432. 10.1007/s10577-013-9366-8 23836161

[B2] Ashida T. Nasuda S. Sato K. Endo T. R. (2007). Dissection of barley chromosome 5H in common wheat. *Genes Genet. Syst.* 82 123–133. 17507778 10.1266/ggs.82.123

[B3] Benito C. Liorente F. Henriques-Gil N. Gallego F. J. Zaragoza C. Delibes A. (1994). A map of rye chromosome 4R with cytological and isozyme markers. *Theor. Appl. Genet.* 87 941–946. 10.1007/BF00225788 24190528

[B4] Berzonsky W. A. Clements R. L. Lafever H. N. (1991). Identification of ‘Amigo’ and ‘Kavkaz’ translocations in Ohio soft red winter wheats (*Triticum aestivum* L.). *Theor. Appl. Genet.* 81 629–634. 10.1007/BF00226729 24221378

[B5] Camacho M. V. Matos M. Gonzales C. Perez-Flores V. Pernauta B. Pinto-Carnida O. (2005). *Secale cereale* inter-microsatellites (SCIMs): chromosomal location and genetic inheritance. *Genetica* 123 303–311. 10.1007/s10709-004-5553-z 15954501

[B6] Chen S. Q. Huang Z. F. Dai Y. Qin Y. Y. Zhang L. L. Gao Y. (2013). The development of 7E chromosome-specific molecular markers for *Thinopyrum elongatum* based on SLAF-seq technology. *PLOS ONE* 8:e65122. 10.1371/journal.pone.0065122 23762296 PMC3677899

[B7] Cuadrado A. Ceoloni C. Jouve N. (1995). Variation in highly repetitive DNA composition of heterochromatin in rye studied by fluorescence in situ hybridization. *Genome* 38 1061–1069. 10.1139/g95-142 18470231

[B8] DeScenzo R. A. Wise R. P. (1996). Variation in the ratio of physical to genetic distance in intervals adjacent to the *Mla* locus on barley chromosome 1H. *Mol. Gen. Genet.* 251 472–482. 10.1007/BF02172376 8709951

[B9] Endo T. R. Gill B. S. (1996). The deletion stocks of common wheat. *J. Hered.* 87 295–307.

[B10] Fu S. L. Chen L. Wang Y. Y. Li M. Yang Z. J. Qiu L. (2015). Oligonucleotide probes for ND-FISH nanlysis to identify rye and wheat chromosomes. *Sci. Rep.* 5:10552. 10.1038/srep10552 25994088 PMC4440213

[B11] Fu S. L. Ren Z. L. Chen X. M. Yan B. J. Tan F. Q. Fu T. H. (2014). New wheat-rye _5_DS-_4_RS⋅_4_RL and _4_RS-_5_DS⋅_5_DL translocation lines with powdery mildew resistance. *J. Plant Res.* 127 743–753. 10.1007/s10265-014-0659-6 25163586

[B12] Fu S. L. Tang Z. X. Ren Z. L. Zhang H. Q. Yan B. J. (2010). Isolation of rye-specific DNA fragment and genetic diversity analysis of rye genus Secale L. using wheat SSR markers. *J. Genet.* 89 489–492. 10.1007/s12041-010-0070-6 21273702

[B13] Gustafson J. P. Ma X. F. Korzum V. Snape J. W. (2009). A consensus map of rye integrating mapping data from five mapping populations. *Theor. Appl. Genet.* 118 793–800. 10.1007/s00122-008-0939-4 19066841

[B14] Gyawali Y. P. Nasuda S. Endo T. R. (2009). Cytological dissection and molecular characterization of chromosome 1R derived from ‘Burgas 2’ common wheat. *Genes Genet. Syst.* 84 407–416. 10.1266/ggs.84.407 20228578

[B15] Gyawali Y. P. Nasuda S. Endo T. R. (2010). A cytological map of the short arm of rye chromosome 1R constructed with 1R dissection stocks of common wheat and PCR-based markers. *Cytogenet. Genome Res.* 129 224–233. 10.1159/000314556 20551617

[B16] Han F. P. Lamb J. C. Birchler J. A. (2006). High frequency of centromere inactivation resulting in stable dicentric chromosomes of maize. *Proc. Natl. Acad. Sci. U.S.A.* 103 3238–3243. 10.1073/pnas.0509650103 16492777 PMC1413895

[B17] Ishihara A. Mizuno N. Islam R. A. K. M. Doležel J. Endo T. R. Nasuda S. (2014). Dissection of barley chromosomes 1H and 6H by the gametocidal system. *Genes Genet. Syst.* 89 203–214. 10.1266/ggs.89.203 25832747

[B18] Kofler R. Bartoš J. Gong L. Stift G. Suchánková P. Šimková H. (2008). Development of microsatellite markers specific for the short arm of rye (*Secale cereale* L.) chromosome 1. *Theor. Appl. Genet.* 117 915–926. 10.1007/s00122-008-0831-2 18626624

[B19] Korzun V. Malyshev S. Kartel N. Westermann T. Weber W. E. Börner A. (1998). A genetic linkage map of rye (*Secale cereale* L.). *Theor. Appl. Genet.* 96 203–208. 10.1007/s001220050728 24197226

[B20] Korzun V. Malyshev S. Voylokov A. V. Börner A. (2001). A genetic map of rye (*Secale cereale* L.) combining RFLP, isozyme, protein, microsatellite and gene loci. *Theor. Appl. Genet.* 102 709–717. 10.1007/s001220051701

[B21] Kumar S. Kumar N. Balyan H. S. Gupta P. K. (2003). 1BL.1RS translocation in some Indian bread wheat genotypes and strategies for its use in future wheat breeding. *Caryologia* 56 23–30. 10.1080/00087114.2003.10589303

[B22] Landjeva S. Korzun V. Tsanev V. Vladova R. Ganeva G. (2006). Distribution of the wheat-rye translocation 1RS.1BL among bread wheat varieties of Bulgaria. *Plant Breed.* 125 102–104. 10.1111/j.1439-0523.2006.01142.x

[B23] Li J. J. Endo T. R. Saito M. Ishikawa G. Nakamura T. Nasuda S. (2013). Homoeologous relationship of rye chromosome arms as detected with wheat PLUG markers. *Chromosoma* 122 555–564. 10.1007/s00412-013-0428-7 23873186

[B24] Li M. Tang Z. X. Qiu L. Wang Y. Y. Tang S. Y. Fu S. L. (2016). Identification and physical mapping of new PCR-based markers specific for the long arm of rye (*Secale cereale* L.) chromosome 6. *J. Genet. Genomics* 43 209–216. 10.1016/j.jgg.2015.11.005 27090607

[B25] Li R. Yu C. Li Y. Lam T. W. Yiu S. M. Kristiansen K. (2009). SOAP2: an improved ultrafast tool for short read alignment. *Bioinformatics* 25 1966–1967. 10.1093/bioinformatics/btp336 19497933

[B26] Lukaszewski A. J. Porter D. R. Baker C. A. Rybka K. Lapinski B. (2001). Attempts to transfer Russion wheat aphid resistance from a rye chromosome in Russian triticales to wheat. *Crop. Sci.* 41 1743–1749. 10.2135/cropsci2001.1743

[B27] Ma X. F. Wanous M. K. Houchins K. Rodriguez Milla M. A. Goicoechea P. G. Wang Z. (2001). Molecular linkage mapping in rye (*Secale cereale* L.). *Theor. Appl. Genet.* 102 517–523. 10.1007/s001220051676

[B28] Milczarski P. Banek-Tabor A. Lebiecka K. Stojałowski S. Myśków B. Masojć P. (2007). New genetic map of rye composed of PCR-based molecular markers and its alignment with the reference map of the DS2 × RXL10 intercross. *J. Appl. Genet.* 48 11–24. 10.1007/BF03194653 17272857

[B29] Milczarski P. Bolibok-Bragoszewska H. Myśków B. Stojatowski S. Heller-Uszyńska K. Góralska M. (2011). A high density consensus map of rye (*Secale cereale* L.) based on DArT markers. *PLOS ONE* 6:e28495. 10.1371/journal.pone.0028495 22163026 PMC3232230

[B30] Milczarski P. Hanek M. Tyka M. Stojalowski S. (2016). The application of GBS markers for extending the dense genetic map of rye (*Secale cereale* L.) and the localization of the *Rfc1* gene restoring male fertility in plants with the C source of sterility-inducing cytoplasm. *J. Appl. Genet.* 57 439–451. 10.1007/s13353-016-0347-4 27085345 PMC5061839

[B31] Nguyen V. Fleury D. Timmins A. Laga H. Hayden M. Mather D. (2015). Addition of rye chromosome 4R to wheat increases anther length and pollen grain number. *Theor. Appl. Genet.* 128 953–964. 10.1007/s00122-015-2482-4 25716820

[B32] Pu J. Wang Q. Shen Y. F. Zhuang L. F. Li C. X. Tan M. F. (2015). Physical mapping of chromosome 4J of *Thinopyrum bessarabicum* using gamma radiation-induced aberrations. *Theor. Appl. Genet.* 128 1319–1328. 10.1007/s00122-015-2508-y 25851001

[B33] Qiu L. Tang Z. X. Li M. Fu S. L. (2016). Development of new PCR-based markers specific for chromosome arms of rye (*Secale cereale* L.). *Genome* 59 159–165. 10.1139/gen-2015-0154 26862664

[B34] Rabinovich S. V. (1998). Importance of wheat-rye translocation for breeding modern cultivars of *Triticum aestivum* L. *Euphytica* 100 323–340. 10.1023/A:1018361819215

[B35] Saal B. Wricke G. (1999). Development of simple sequence repeat markers in rye (*Secale cereale* L.). *Genome* 42 964–972. 10.1139/g99-052 10584314

[B36] Sakata M. Nasuda S. Endo T. R. (2010). Dissection of barley chromosome 4H in common wheat by the gametocidal system and cytological mapping of chromosome 4H with EST markers. *Genes Genet. Syst.* 85 19–29. 10.1266/ggs.85.19 20410662

[B37] Schneider A. Rakszegi M. Molnár-Láng M. Szakács É (2016). Production and cytomolecular identification of new wheat-perennial rye (*Secale cereale*) disomic addition lines with yellow rust resistance (6R) and increased arabinoxylan and protein content (1R, 4R, 6R). *Theor. Appl. Genet.* 129 1045–1059. 10.1007/s00122-016-2682-6 26883040

[B38] Song L. Q. Lu Y. Q. Zhang J. P. Pan C. L. Yang X. M. Li X. Q. (2016). Physical mapping of *Agropyron cristatum* chromosome 6P using deletion lines in common wheat background. *Theor. Appl. Genet.* 129 1023–1034. 10.1007/s00122-016-2680-8 26920547

[B39] Tang Z. X. Yang Z. J. Fu S. L. (2014). Oligonucleotides replacing the roles of repetitive sequences pAs1, pSc119.2, pTa-535, pTa71, CCS1, and pAWRC.1 for FISH analysis. *J. Appl. Genet.* 55 313–318. 10.1007/s13353-014-0215-z 24782110

[B40] Tomita M. Seno A. (2012). Rye chromosome-specific polymerase chain reaction products developed by primers designed from the *EcoO109I* recognition site. *Genome* 55 370–382. 10.1139/g2012-024 22563759

[B41] Tsuchida M. Fukushima T. Nasuda S. Masoudi-Nejad A. Ishikawa G. Nakamura T. (2008). Dissection of rye chromosome 1R in common wheat. *Genes Genet. Syst.* 83 43–53. 10.1266/ggs.83.43 18379133

[B42] Xu H. Yin D. Li L. Wang Q. Li X. Yang X. (2012). Development and application of EST-based markers specific for chromosome arms of rye (*Secale cereale* L.). *Cytogenet*. *Genome Res.* 136 220–228. 10.1159/000336478 22354334

[B43] Zhuang L. F. Liu P. Liu Z. Q. Chen T. T. Wu N. Sun L. (2015). Multiple structural aberrations and physical mapping of rye chromosome 2R introgressed into wheat. *Mol. Breed.* 35 133.

