# Supplementary material for: Physical Location of New PCR-Based Markers and Powdery Mildew Resistance Gene(s) on Rye (Secale cereale L.) Chromosome 4 Using 4R Dissection Lines
Source: Front Plant Sci. 2017 Oct 10;8:1716. doi: 10.3389/fpls.2017.01716 (PMC5641395; doi:10.3389/fpls.2017.01716)
Supplement: Supplementary file 2 [file Table_2.DOC]

**Supplementary Table 2** Location of 4R^Ku^-specific markers on six regions of 4R^Ku^ chromosome using 4R^Ku^ dissection lines*

| **Marker** | **4R** | **4RS** | **16T197-6**  **(**4RS-5DS.5DL**)** | **16T175-1**  **(**5DS.4RS) | **4RL** | **16T177-4**  (broken 5DS-4RS.4RL; Break point on 4RL is between the centromere and pSc119.2 signal site) | **16T196-22**  (broken 5DS-4RS.4RL; Break point on 4RL is at the pSc119.2 signal site ) | **16T75-24**  (5BS.5BL-4RL) |
| --- | --- | --- | --- | --- | --- | --- | --- | --- |
| KU-4R.004 | + |  |  |  | + |  |  | + |
| KU-4R.009 | + |  |  |  | + |  |  | + |
| KU-4R.011 | + |  |  |  | + |  |  | + |
| KU-4R.012 | + |  |  |  | + |  |  |  |
| KU-4R.021 | + | + | + |  |  |  |  |  |
| KU-4R.022 | + | + | + |  |  |  |  |  |
| KU-4R.024 | + | + | + |  |  |  |  |  |
| KU-4R.025 | + | + | + |  |  |  |  |  |
| KU-4R.026 | + | + | + |  |  |  |  |  |
| KU-4R.030 | + | + | + |  |  |  |  |  |
| KU-4R.037 | + | + | + |  |  |  |  |  |
| KU-4R.045 | + | + | + |  |  |  |  |  |
| KU-4R.050 | + |  |  |  | + |  |  | + |
| KU-4R.053 | + |  |  |  | + | + | + |  |
| KU-4R.057 | + |  |  |  | + |  |  | + |
| KU-4R.067 | + | + |  |  |  |  |  |  |
| **Marker** | **4R** | **4RS** | **16T197-6**  (4RS-5DS.5DL) | **16T175-1**  **(**5DS.4RS) | **4RL** | **16T177-4**  (broken 5DS-4RS.4RL; Break point on 4RL is between the centromere and pSc119.2 signal site) | **16T-196-22**  (broken 5DS-4RS.4RL; Break point on 4RL is at the pSc119.2 signal site ) | **16T75-24**  (5BS.5BL-4RL) |
| KU-4R.068 | + | + | + |  |  |  |  |  |
| KU-4R.069 | + |  |  |  | + | + | + |  |
| KU-4R.071 | + |  |  |  | + |  |  | + |
| KU-4R.078 | + |  |  |  | + |  |  | + |
| KU-4R.081 | + |  |  |  | + |  |  | + |
| KU-4R.083 | + |  |  |  | + | + | + |  |
| KU-4R.087 | + |  |  |  | + | + | + |  |
| KU-4R.089 | + |  |  |  | + |  |  | + |
| KU-4R.095 | + | + | + |  |  |  |  |  |
| KU-4R.104 | + | + | + |  |  |  |  |  |
| KU-4R.107 | + |  |  |  | + |  |  | + |
| KU-4R.111 | + |  |  |  | + |  |  | + |
| KU-4R.114 | + | + | + |  |  |  |  |  |
| KU-4R.115 | + | + |  | + |  | + | + |  |
| KU-4R.116 | + |  |  |  | + |  |  | + |
| KU-4R.117 | + |  |  |  | + |  |  | + |
| KU-4R.118 | + |  |  |  | + |  |  | + |
| KU-4R.121 | + |  |  |  | + |  | + | + |
| KU-4R.122 | + |  |  |  | + | + | + |  |
| KU-4R.130 | + |  |  |  | + |  |  | + |
| **Marker** | **4R** | **4RS** | **16T197-6**  **(**4RS-5DS.5DL**)** | **16T175-1**  **(**5DS.4RS mini) | **4RL** | **16T177-4**  (broken 5DS-4RS.4RL; Break point on 4RL is between the centromere and pSc119.2 signal site) | **16T-196-22**  (broken 5DS-4RS.4RL; Break point on 4RL is at the pSc119.2 signal site ) | **16T75-24**  (5BS.5BL-4RL) |
| KU-4R.135 | + | + | + |  |  |  |  |  |
| KU-4R.136 | + |  |  |  | + |  |  | + |
| KU-4R.137 | + |  |  |  | + |  | + | + |
| KU-4R.142 | + |  |  |  | + |  |  | + |
| KU-4R.143 | + |  |  |  | + |  | + | + |
| KU-4R.144 | + | + | + |  |  |  |  |  |
| KU-4R.146 | + |  |  |  | + |  |  | + |
| KU-4R.149 | + |  |  |  | + | + | + |  |
| KU-4R.154 | + |  |  |  | + |  |  | + |
| KU-4R.155 | + |  |  |  | + |  |  | + |
| KU-4R.167 | + |  |  |  | + |  |  | + |
| KU-4R.170 | + | + | + |  |  |  |  |  |
| KU-4R.175 | + |  |  |  | + |  |  | + |
| KU-4R.176 | + | + | + |  |  |  |  |  |
| KU-4R.177 | + | + | + |  |  |  |  |  |
| KU-4R.182 | + |  |  |  | + |  | + | + |
| KU-4R.183 | + |  |  |  | + |  |  | + |
| KU-4R.184 | + |  |  |  | + |  |  | + |
| KU-4R.185 | + | + | + |  |  |  |  |  |
| KU-4R.186 | + |  |  |  | + |  |  | + |
| **Marker** | **4R** | **4RS** | **16T197-6**  **(**4RS-5DS.5DL**)** | **16T175-1**  **(**5DS.4RS) | **4RL** | **16T177-4**  (broken 5DS-4RS.4RL; Break point on 4RL is between the centromere and pSc119.2 signal site) | **16T-196-22**  (broken 5DS-4RS.4RL; Break point on 4RL is at the pSc119.2 signal site ) | **16T75-24**  (5BS.5BL-4RL) |
| KU-4R.188 | + | + |  |  |  |  |  |  |
| KU-4R.197 | + |  |  |  | + |  |  | + |
| KU-4R.200 | + |  |  |  | + |  |  | + |
| KU-4R.201 | + | + | + |  |  |  |  |  |
| KU-4R.204 | + | + | + |  |  |  |  |  |
| KU-4R.205 | + | + | + |  |  |  |  |  |
| KU-4R.206 | + |  |  |  | + |  |  | + |
| KU-4R.207 | + | + | + |  |  |  |  |  |
| KU-4R.211 | + | + | + |  |  |  |  |  |
| KU-4R.216 | + | + | + |  |  |  |  |  |
| KU-4R.217 | + | + | + |  |  |  |  |  |
| KU-4R.222 | + | + | + |  |  |  |  |  |
| KU-4R.224 | + | + | + |  |  |  |  |  |
| KU-4R.226 | + |  |  |  | + |  |  | + |
| KU-4R.228 | + |  |  |  | + |  |  | + |
| KU-4R.231 | + | + | + |  |  |  |  |  |
| KU-4R.233 | + | + | + |  |  |  |  |  |
| KU-4R.234 | + | + | + |  |  |  |  |  |
| KU-4R.235 | + |  |  |  | + |  |  | + |
| KU-4R.236 | + | + | + |  |  |  |  |  |
| **Marker** | **4R** | **4RS** | **16T197-6**  **(**4RS-5DS.5DL**)** | **16T175-1**  **(**5DS.4RS ) | **4RL** | **16T177-4**  (broken 5DS-4RS.4RL; Break point on 4RL is between the centromere and pSc119.2 signal site) | **16T-196-22**  (broken 5DS-4RS.4RL; Break point on 4RL is at the pSc119.2 signal site ) | **16T75-24**  (5BS.5BL-4RL) |
| KU-4R.240 | + | + | + |  |  |  |  |  |
| KU-4R.249 | + | + | + |  |  |  |  |  |
| KU-4R.250 | + | + | + |  |  |  |  |  |
| KU-4R.256 | + | + | + |  |  |  |  |  |
| KU-4R.258 | + | + | + |  |  |  |  |  |
| KU-4R.262 | + |  |  |  | + | + | + |  |
| KU-4R.264 | + | + | + |  |  |  |  |  |
| KU-4R.267 | + |  |  |  | + | + | + |  |
| KU-4R.268 | + |  |  |  | + |  |  | + |
| KU-4R.274 | + |  |  |  | + |  |  | + |
| KU-4R.295 | + |  |  |  | + | + | + |  |
| KU-4R.296 | + | + | + |  |  |  |  |  |
| KU-4R.299 | + | + | + |  |  |  |  |  |
| KU-4R.303 | + | + | + |  |  |  |  |  |
| KU-4R.309 | + |  |  |  | + |  | + | + |
| KU-4R.310 | + |  |  |  | + |  |  | + |
| KU-4R.311 | + | + | + |  |  |  |  |  |
| KU-4R.312 | + |  |  |  | + |  |  | + |
| KU-4R.314 | + |  |  |  | + | + | + |  |
| KU-4R.317 | + | + |  |  |  |  |  |  |
| **Marker** | **4R** | **4RS** | **16T197-6**  **(**4RS-5DS.5DL**)** | **16T175-1**  **(**5DS.4RS mini) | **4RL** | **16T177-4**  (broken 5DS-4RS.4RL; Break point on 4RL is between the centromere and pSc119.2 signal site) | **16T-196-22**  (broken 5DS-4RS.4RL; Break point on 4RL is at the pSc119.2 signal site ) | **16T75-24**  (5BS.5BL-4RL) |
| KU-4R.327 | + | + | + |  |  |  |  |  |
| KU-4R.328 | + | + | + |  |  |  |  |  |
| KU-4R.329 | + | + | + |  |  |  |  |  |
| KU-4R.330 | + |  |  |  | + |  | + |  |
| KU-4R.333 | + |  |  |  | + |  |  | + |
| KU-4R.334 | + |  |  |  | + |  | + | + |
| KU-4R.337 | + |  |  |  | + |  | + | + |
| KU-4R.338 | + |  |  |  | + |  | + | + |
| KU-4R.346 | + |  |  |  | + | + | + |  |
| KU-4R.348 | + |  |  |  | + |  |  | + |
| KU-4R.350 | + |  |  |  | + |  |  | + |
| KU-4R.351 | + |  |  |  | + |  |  | + |
| KU-4R.353 | + |  |  |  | + |  | + | + |
| KU-4R.354 | + |  |  |  | + |  |  | + |
| KU-4R.356 | + | + | + |  |  |  |  |  |
| KU-4R.361 | + |  |  |  | + |  |  | + |
| KU-4R.363 | + |  |  |  | + |  |  | + |
| KU-4R.367 | + |  |  |  | + | + | + |  |
| KU-4R.368 | + | + | + |  |  |  |  |  |
| KU-4R.370 | + |  |  |  | + | + | + |  |
| **Marker** | **4R** | **4RS** | **16T197-6**  **(**4RS-5DS.5DL**)** | **16T175-1**  **(**5DS.4RS ) | **4RL** | **16T177-4**  (broken 5DS-4RS.4RL; Break point on 4RL is between the centromere and pSc119.2 signal site) | **16T-196-22**  (broken 5DS-4RS.4RL; Break point on 4RL is at the pSc119.2 signal site ) | **16T75-24**  (5BS.5BL-4RL) |
| KU-4R.373 | + | + | + |  |  |  |  |  |
| KU-4R.378 | + | + |  | + |  | + | + |  |
| KU-4R.384 | + |  |  |  | + |  |  | + |
| KU-4R.387 | + | + | + |  |  |  |  |  |
| KU-4R.390 | + |  |  |  | + |  |  | + |
| KU-4R.393 | + | + |  | + |  | + | + |  |
| KU-4R.394 | + | + | + |  |  |  |  |  |
| KU-4R.397 | + | + | + |  |  |  |  |  |
| KU-4R.403 | + |  |  |  | + |  |  | + |
| KU-4R.404 | + |  |  |  | + |  | + | + |
| KU-4R.405 | + | + |  | + |  | + | + |  |
| KU-4R.406 | + |  |  |  | + |  |  | + |
| KU-4R.407 | + |  |  |  | + | + | + |  |
| KU-4R.408 | + |  |  |  | + |  |  | + |
| KU-4R.412 | + |  |  |  | + |  | + | + |
| KU-4R.413 | + | + | + |  |  |  |  |  |
| KU-4R.416 | + |  |  |  | + |  | + | + |
| KU-4R.418 | + | + | + |  |  |  |  |  |
| KU-4R.420 | + | + | + |  |  |  |  |  |
| KU-4R.421 | + | + | + |  |  |  |  |  |
| **Marker** | **4R** | **4RS** | **16T197-6**  **(**4RS-5DS.5DL**)** | **16T175-1**  **(**5DS.4RS ) | **4RL** | **16T177-4**  (broken 5DS-4RS.4RL; Break point on 4RL is between the centromere and pSc119.2 signal site) | **16T-196-22**  (broken 5DS-4RS.4RL; Break point on 4RL is at the pSc119.2 signal site ) | **16T75-24**  (5BS.5BL-4RL) |
| KU-4R.422 | + |  |  |  | + |  |  | + |
| KU-4R.423 | + |  |  |  | + |  |  | + |
| KU-4R.424 | + |  |  |  | + | + | + |  |
| KU-4R.426 | + |  |  |  | + | + | + |  |
| KU-4R.427 | + | + | + |  |  |  |  |  |
| KU-4R.428 | + | + |  |  |  |  |  |  |
| KU-4R.429 | + | + |  |  |  |  |  |  |
| KU-4R.430 | + | + | + |  |  |  |  |  |
| KU-4R.431 | + | + | + |  |  |  |  |  |
| KU-4R.432 | + |  |  |  | + |  |  | + |
| KU-4R.433 | + |  |  |  | + |  | + | + |
| KU-4R.437 | + | + |  |  |  |  |  |  |
| KU-4R.441 | + | + | + |  |  |  |  |  |
| KU-4R.443 | + |  |  |  | + | + | + |  |
| KU-4R.446 | + |  |  |  | + |  |  | + |
| KU-4R.449 | + | + | + |  |  |  |  |  |
| KU-4R.451 | + |  |  |  | + |  | + | + |
| KU-4R.454 | + |  |  |  | + |  |  | + |
| KU-4R.455 | + |  |  |  | + |  |  | + |
| **Marker** | **4R** | **4RS** | **16T197-6**  **(**4RS-5DS.5DL**)** | **16T175-1**  **(**5DS.4RS) | **4RL** | **16T177-4**  (broken 5DS-4RS.4RL; Break point on 4RL is between the centromere and pSc119.2 signal site) | **16T-196-22**  (broken 5DS-4RS.4RL; Break point on 4RL is at the pSc119.2 signal site ) | **16T75-24**  (5BS.5BL-4RL) |
| KU-4R.456 | + |  |  |  | + |  |  | + |
| KU-4R.459 | + | + | + |  |  |  |  |  |
| KU-4R.460 | + | + |  |  |  |  |  |  |
| KU-4R.461 | + |  |  |  | + |  |  | + |
| KU-4R.465 | + |  |  |  | + |  |  | + |
| KU-4R.467 | + |  |  |  | + |  |  | + |
| KU-4R.468 | + | + | + |  |  |  |  |  |
| KU-4R.470 | + |  |  |  | + | + | + |  |
| KU-4R.471 | + |  |  |  | + |  |  | + |
| KU-4R.473 | + |  |  |  | + |  | + | + |
| KU-4R.474 | + |  |  |  | + |  |  | + |
| KU-4R.475 | + |  |  |  | + | + | + |  |
| KU-4R.478 | + |  |  |  | + | + | + |  |
| KU-4R.479 | + |  |  |  | + |  |  | + |
| KU-4R.480 | + |  |  |  | + |  |  | + |
| KU-4R.481 | + | + | + |  |  |  |  |  |
| KU-4R.482 | + |  |  |  | + |  |  | + |
| KU-4R.485 | + |  |  |  | + |  |  | + |
| KU-4R.486 | + |  |  |  | + |  |  | + |
| **Marker** | **4R** | **4RS** | **16T197-6**  **(**4RS-5DS.5DL | **16T175-1**  **(**5DS.4RS) | **4RL** | **16T177-4**  (broken 5DS-4RS.4RL; Break point on 4RL is between the centromere and pSc119.2 signal site) | **16T-196-22**  (broken 5DS-4RS.4RL; Break point on 4RL is at the pSc119.2 signal site ) | **16T75-24**  (5BS.5BL-4RL) |
| KU-4R.491 | + |  |  |  | + |  | + | + |
| KU-4R.492 | + | + | + |  |  |  |  |  |
| KU-4R.494 | + |  |  |  | + |  |  | + |
| KU-4R.495 | + | + | + |  |  |  |  |  |
| KU-4R.498 | + |  |  |  | + |  | + | + |
| KU-4R.499 | + |  |  |  | + | + | + |  |
| KU-4R.502 | + |  |  |  | + |  |  | + |
| KU-4R.506 | + |  |  |  | + |  |  | + |
| KU-4R.508 | + | + |  | + |  | + | + |  |
| KU-4R.509 | + |  |  |  | + | + | + |  |
| KU-4R.510 | + |  |  |  | + |  |  | + |
| KU-4R.511 | + |  |  |  | + |  | + | + |
| KU-4R.513 | + | + | + |  |  |  |  |  |
| KU-4R.514 | + | + | + |  |  |  |  |  |
| KU-4R.516 | + |  |  |  | + |  |  | + |
| KU-4R.519 | + |  |  |  | + |  |  | + |
| KU-4R.521 | + |  |  |  | + |  |  | + |
| KU-4R.523 | + | + | + |  |  |  |  |  |
| KU-4R.524 | + |  |  |  | + |  |  | + |
| **Marker** | **4R** | **4RS** | **16T197-6**  **(**4RS-5DS.5DL**)** | **16T175-1**  **(**5DS.4RS) | **4RL** | **16T177-4**  (broken 5DS-4RS.4RL; Break point on 4RL is between the centromere and pSc119.2 signal site) | **16T-196-22**  (broken 5DS-4RS.4RL; Break point on 4RL is at the pSc119.2 signal site ) | **16T75-24**  (5BS.5BL-4RL) |
| KU-4R.525 | + |  |  |  | + |  |  | + |
| KU-4R.529 | + | + |  | + |  | + | + |  |
| KU-4R.532 | + | + | + |  |  |  |  |  |
| KU-4R.533 | + |  |  |  | + |  |  | + |
| KU-4R.540 | + |  |  |  | + |  | + |  |
| KU-4R.542 | + |  |  |  | + |  |  |  |
| KU-4R.544 | + |  |  |  | + |  |  | + |
| KU-4R.548 | + |  |  |  | + |  | + | + |
| KU-4R.549 | + | + | + |  |  |  |  |  |
| KU-4R.551 | + |  |  |  | + | + | + |  |
| KU-4R.552 | + | + | + |  |  |  |  |  |
| KU-4R.554 | + | + | + |  |  |  |  |  |
| KU-4R.555 | + | + |  |  |  |  |  |  |
| KU-4R.556 | + | + | + |  |  |  |  |  |
| KU-4R.559 | + |  |  |  | + | + | + |  |
| KU-4R.562 | + | + | + |  |  |  |  |  |
| KU-4R.563 | + |  |  |  | + |  | + |  |
| KU-4R.564 | + |  |  |  | + | + | + |  |
| **Marker** | **4R** | **4RS** | **16T197-6**  **(**4RS-5DS.5DL**)** | **16T175-1**  **(**5DS.4RS) | **4RL** | **16T177-4**  (broken 5DS-4RS.4RL; Break point on 4RL is between the centromere and pSc119.2 signal site) | **16T-196-22**  (broken 5DS-4RS.4RL; Break point on 4RL is at the pSc119.2 signal site ) | **16T75-24**  (5BS.5BL-4RL) |
| KU-4R.567 | + | + | + |  |  |  |  |  |
| KU-4R.568 | + | + | + |  |  |  |  |  |
| KU-4R.571 | + |  |  |  | + | + | + |  |
| KU-4R.573 | + |  |  |  | + |  |  | + |
| KU-4R.575 | + | + | + |  |  |  |  |  |
| KU-4R.576 | + | + | + |  |  |  |  |  |
| KU-4R.578 | + |  |  |  | + |  |  | + |
| KU-4R.579 | + |  |  |  | + |  |  | + |
| KU-4R.589 | + | + |  | + |  | + | + |  |
| KU-4R.596 | + |  |  |  | + |  |  | + |
| KU-4R.597 | + |  |  |  | + |  | + |  |
| KU-4R.598 | + | + | + |  |  |  |  |  |
| KU-4R.599 | + | + | + |  |  |  |  |  |
| KU-4R.600 | + |  |  |  | + |  | + | + |
| KU-4R.601 | + |  |  |  | + | + | + |  |
| KU-4R.603 | + | + | + |  |  |  |  |  |
| KU-4R.604 | + | + | + |  |  |  |  |  |
| KU-4R.608 | + |  |  |  | + |  |  | + |
| **Marker** | **4R** | **4RS** | **16T197-6**  **(**4RS-5DS.5DL**)** | **16T175-1**  **(**5DS.4RS) | **4RL** | **16T177-4**  (broken 5DS-4RS.4RL; Break point on 4RL is between the centromere and pSc119.2 signal site) | **16T-196-22**  (broken 5DS-4RS.4RL; Break point on 4RL is at the pSc119.2 signal site ) | **16T75-24**  (5BS.5BL-4RL) |
| KU-4R.609 | + | + | + |  |  |  |  |  |
| KU-4R.610 | + |  |  |  | + |  |  | + |
| KU-4R.611 | + |  |  |  | + | + | + |  |
| KU-4R.612 | + |  |  |  | + |  | + | + |
| KU-4R.614 | + |  |  |  | + | + | + |  |
| KU-4R.615 | + |  |  |  | + |  |  | + |
| KU-4R.617 | + |  |  |  | + |  |  | + |
| KU-4R.619 | + | + | + |  |  |  |  |  |
| KU-4R.620 | + | + |  | + |  | + | + |  |
| KU-4R.622 | + | + | + |  |  |  |  |  |
| KU-4R.624 | + |  |  |  | + |  |  | + |
| KU-4R.626 | + |  |  |  | + |  |  | + |
| KU-4R.627 | + |  |  |  | + | + | + |  |
| KU-4R.628 | + |  |  |  | + |  |  |  |
| KU-4R.632 | + | + | + |  |  |  |  |  |
| KU-4R.636 | + |  |  |  | + |  |  | + |
| KU-4R.638 | + |  |  |  | + |  |  | + |
| KU-4R.641 | + |  |  |  | + |  |  | + |
| **Marker** | **4R** | **4RS** | **16T197-6**  **(**4RS-5DS.5DL**)** | **16T175-1**  **(**5DS.4RS) | **4RL** | **16T177-4**  (broken 5DS-4RS.4RL; Break point on 4RL is between the centromere and pSc119.2 signal site) | **16T-196-22**  (broken 5DS-4RS.4RL; Break point on 4RL is at the pSc119.2 signal site ) | **16T75-24**  (5BS.5BL-4RL) |
| KU-4R.642 | + |  |  |  | + | + | + |  |
| KU-4R.643 | + |  |  |  | + |  |  |  |
| KU-4R.644 | + |  |  |  | + |  |  | + |
| KU-4R.651 | + |  |  |  | + |  |  | + |
| KU-4R.652 | + |  |  |  | + |  |  | + |
| KU-4R.654 | + |  |  |  | + | + | + |  |
| KU-4R.655 | + | + | + |  |  |  |  |  |
| KU-4R.657 | + |  |  |  | + |  |  | + |
| KU-4R.661 | + |  |  |  | + |  | + | + |
| KU-4R.662 | + |  |  |  | + | + | + |  |
| KU-4R.663 | + | + | + |  |  |  |  |  |
| KU-4R.664 | + | + |  | + |  | + | + |  |
| KU-4R.668 | + | + | + |  |  |  |  |  |
| KU-4R.669 | + |  |  |  | + | + | + |  |
| KU-4R.676 | + | + | + |  |  |  |  |  |
| KU-4R.678 | + | + | + |  |  |  |  |  |
| KU-4R.679 | + |  |  |  | + | + | + |  |
| KU-4R.682 | + | + | + |  |  |  |  |  |
| **Marker** | **4R** | **4RS** | **16T197-6**  **(**4RS-5DS.5DL**)** | **16T175-1**  **(**5DS.4RS) | **4RL** | **16T177-4**  (broken 5DS-4RS.4RL; Break point on 4RL is between the centromere and pSc119.2 signal site) | **16T-196-22**  (broken 5DS-4RS.4RL; Break point on 4RL is at the pSc119.2 signal site ) | **16T75-24**  (5BS.5BL-4RL) |
| KU-4R.686 | + | + | + |  |  |  |  |  |
| KU-4R.687 | + |  |  |  | + |  |  |  |
| KU-4R.696 | + | + | + |  |  |  |  |  |
| KU-4R.698 | + | + | + |  |  |  |  |  |
| KU-4R.700 | + |  |  |  | + |  |  | + |
| KU-4R.705 | + | + | + |  |  |  |  |  |
| KU-4R.706 | + |  |  |  | + | + | + |  |
| KU-4R.707 | + |  |  |  | + |  |  | + |
| KU-4R.708 | + |  |  |  | + |  |  | + |
| KU-4R.709 | + |  |  |  | + |  |  | + |
| KU-4R.710 | + | + | + |  |  |  |  |  |
| KU-4R.711 | + | + | + |  |  |  |  |  |
| KU-4R.714 | + |  |  |  | + |  | + | + |
| KU-4R.724 | + |  |  |  | + |  |  | + |
| KU-4R.727 | + | + | + |  |  |  |  |  |
| KU-4R.732 | + | + |  |  |  |  |  |  |
| KU-4R.733 | + | + | + |  |  |  |  |  |
| KU-4R.735 | + |  |  |  | + |  |  | + |
| **Marker** | **4R** | **4RS** | **16T197-6**  **(**4RS-5DS.5DL**)** | **16T175-1**  **(**5DS.4RS) | **4RL** | **16T177-4**  (broken 5DS-4RS.4RL; Break point on 4RL is between the centromere and pSc119.2 signal site) | **16T-196-22**  (broken 5DS-4RS.4RL; Break point on 4RL is at the pSc119.2 signal site ) | **16T75-24**  (5BS.5BL-4RL) |
| KU-4R.736 | + | + | + |  |  |  |  |  |
| KU-4R.739 | + | + | + |  |  |  |  |  |
| KU-4R.740 | + |  |  |  | + | + | + |  |
| KU-4R.743 | + |  |  |  | + |  |  | + |
| KU-4R.745 | + | + | + |  |  |  |  |  |
| KU-4R.747 | + | + | + |  |  |  |  |  |
| KU-4R.749 | + | + | + |  |  |  |  |  |
| KU-4R754 | + |  |  |  | + |  |  | + |
| KU-4R.755 | + |  |  |  | + |  |  | + |
| KU-4R.763 | + | + | + |  |  |  |  |  |
| KU-4R.764 | + | + | + |  |  |  |  |  |
| KU-4R.774 | + | + | + |  |  |  |  |  |
| KU-4R.777 | + | + | + |  |  |  |  |  |
| KU-4R.781 | + |  |  |  | + |  | + | + |
| KU-4R.785 | + | + |  | + |  | + | + |  |
| KU-4R.788 | + | + | + |  |  |  |  |  |
| KU-4R.793 | + |  |  |  | + |  |  | + |
| KU-4R.787 | + |  |  |  | + |  |  | + |
| **Marker** | **4R** | **4RS** | **16T197-6**  **(**4RS-5DS.5DL**)** | **16T175-1**  **(**5DS.4RS) | **4RL** | **16T177-4**  (broken 5DS-4RS.4RL; Break point on 4RL is between the centromere and pSc119.2 signal site) | **16T-196-22**  (broken 5DS-4RS.4RL; Break point on 4RL is at the pSc119.2 signal site ) | **16T75-24**  (5BS.5BL-4RL) |
| KU.5 | + | + |  |  |  |  |  |  |
| KU.53 | + | + | ＋ |  |  |  |  |  |
| KU.59 | + | + | ＋ |  |  |  |  |  |
| KU.132 | + | + |  | + |  | + | + |  |
| KU.172 | + | + | ＋ |  |  |  |  |  |
| KU.261 | + | + | ＋ |  |  |  |  |  |
| KU.280 | + | + | ＋ |  |  |  |  |  |
| KU.428 | + | ＋ |  |  |  |  |  |  |
| KU.465 | + | + |  |  |  |  |  |  |
| KU.488 | + | + |  | + |  | + | + |  |
| KU.515 | + | + |  |  |  |  |  |  |
| KU.564 | + | + | ＋ |  |  |  |  |  |
| KU.648 | + | + | ＋ |  |  |  |  |  |
| KU.663 | + | + | ＋ |  |  |  |  |  |
| KU.737 | + | + | ＋ |  |  |  |  |  |
| KU.771 | + | + | ＋ |  |  |  |  |  |
| KU.818 | + | + |  |  |  |  |  |  |
| KU.837 | + | + | ＋ |  |  |  |  |  |
| KU.838 | + | + |  |  |  |  |  |  |
| KU.839 | + | + |  |  |  |  |  |  |
| **Marker** | **4R** | **4RS** | **16T197-6**  **(**4RS-5DS.5DL**)** | **16T175-1**  **(**5DS.4RS) | **4RL** | **16T177-4**  (broken 5DS-4RS.4RL; Break point on 4RL is between the centromere and pSc119.2 signal site) | **16T-196-22**  (broken 5DS-4RS.4RL; Break point on 4RL is at the pSc119.2 signal site ) | **16T75-24**  (5BS.5BL-4RL) |
| KU.858 | + | + | ＋ |  |  |  |  |  |
| KU.879 | + | ＋ | ＋ |  |  |  |  |  |
| KU.960 | + | + | ＋ |  |  |  |  |  |
| KU.973 | + | + | ＋ |  |  |  |  |  |
| KU.1004 | + | + |  | + |  | + | + |  |
| KU.1013 | + | + |  | + |  | + | + |  |
| KU.1036 | + | + | ＋ |  |  |  |  |  |
| KU.1041 | + | + |  | + |  | + | + |  |
| KU.1053 | + | ＋ | ＋ |  |  |  |  |  |
| KU.1054 | + | ＋ | ＋ |  |  |  |  |  |
| KU.1068 | + | ＋ | ＋ |  |  |  |  |  |
| KU.18 | + |  |  |  | + |  |  | + |
| KU.29 | + |  |  |  | + |  |  |  |
| KU.42 | + |  |  |  | + |  |  |  |
| KU.49 | + |  |  |  | + |  |  |  |
| KU.56 | + |  |  |  | + |  |  |  |
| KU.58 | + |  |  |  | + |  |  |  |
| KU.93 | + |  |  |  | + |  |  |  |
| KU.144 | + |  |  |  | + |  |  |  |
| KU.158 | + |  |  |  | + | + | + |  |
| **Marker** | **4R** | **4RS** | **16T197-6**  **(**4RS-5DS.5DL**)** | **16T175-1**  **(**5DS.4RS) | **4RL** | **16T177-4**  (broken 5DS-4RS.4RL; Break point on 4RL is between the centromere and pSc119.2 signal site) | **16T-196-22**  (broken 5DS-4RS.4RL; Break point on 4RL is at the pSc119.2 signal site ) | **16T75-24**  (5BS.5BL-4RL) |
| KU.170 | + |  |  |  | + |  |  |  |
| KU.183 | + |  |  |  | + |  |  |  |
| KU.187 | + |  |  |  | + |  |  |  |
| KU.240 | + |  |  |  | + |  |  |  |
| KU.242 | + |  |  |  | + |  |  |  |
| KU.259 | + |  |  |  | + | + | + |  |
| KU.319 | + |  |  |  | + |  |  | + |
| KU.338 | + |  |  |  | + |  |  | + |
| KU.346 | + |  |  |  | + | + | + |  |
| KU.347 | + |  |  |  | + |  |  |  |
| KU.368 | + |  |  |  | + |  |  |  |
| KU.374 | + |  |  |  | + |  |  |  |
| KU.386 | + |  |  |  | + |  |  |  |
| KU.391 | + |  |  |  | + |  | + |  |
| KU.422 | + |  |  |  | + |  |  |  |
| KU.447 | + |  |  |  | + |  |  |  |
| KU.459 | + |  |  |  | + |  |  | + |
| KU.468 | + |  |  |  | + |  |  |  |
| KU.482 | + |  |  |  | + |  |  | + |
| KU.543 | + |  |  |  | + |  |  | + |
| **Marker** | **4R** | **4RS** | **16T197-6**  **(**4RS-5DS.5DL**)** | **16T175-1**  **(**5DS.4RS) | **4RL** | **16T177-4**  (broken 5DS-4RS.4RL; Break point on 4RL is between the centromere and pSc119.2 signal site) | **16T-196-22**  (broken 5DS-4RS.4RL; Break point on 4RL is at the pSc119.2 signal site ) | **16T75-24**  (5BS.5BL-4RL) |
| KU.568 | + |  |  |  | ＋ |  |  |  |
| KU.572 | + |  |  |  | ＋ |  |  |  |
| KU.582 | + |  |  |  | + | + | + |  |
| KU.586 | + |  |  |  | + |  |  | + |
| KU.594 | + |  |  |  | + |  |  |  |
| KU.610 | + |  |  |  | + | + | + |  |
| KU.614 | + |  |  |  | + |  |  |  |
| KU.631 | + |  |  |  | + | + | + |  |
| KU.632 | + |  |  |  | + |  |  |  |
| KU.679 | + |  |  |  | + |  |  |  |
| KU.687 | + |  |  |  | + |  |  |  |
| KU.706 | + |  |  |  | + | + | + |  |
| KU.708 | + |  |  |  | + |  |  |  |
| KU.715 | + |  |  |  | + |  |  |  |
| KU.720 | + |  |  |  | + |  |  |  |
| KU.732 | + |  |  |  | + |  | + |  |
| KU.733 | + |  |  |  | + |  |  | + |
| KU.756 | + |  |  |  | + |  |  |  |
| KU.790 | + |  |  |  | + |  |  |  |
| KU.799 | + |  |  |  | + |  |  |  |
| **Marker** | **4R** | **4RS** | **16T197-6**  **(**4RS-5DS.5DL**)** | **16T175-1**  **(**5DS.4RS) | **4RL** | **16T177-4**  (broken 5DS-4RS.4RL; Break point on 4RL is between the centromere and pSc119.2 signal site) | **16T-196-22**  (broken 5DS-4RS.4RL; Break point on 4RL is at the pSc119.2 signal site ) | **16T75-24**  (5BS.5BL-4RL) |
| KU.812 | + |  |  |  | + |  |  |  |
| KU.828 | + |  |  |  | + |  |  |  |
| KU.830 | + |  |  |  | + |  |  |  |
| KU.847 | + |  |  |  | + |  |  | + |
| KU.848 | + |  |  |  | + |  |  |  |
| KU.861 | + |  |  |  | + | + | + |  |
| KU.867 | + |  |  |  | + |  |  |  |
| KU.888 | + |  |  |  | + |  |  |  |
| KU.892 | + |  |  |  | + |  |  | + |
| KU.900 | + |  |  |  | + |  |  |  |
| KU.922 | + |  |  |  | + | + | + |  |
| KU.926 | + |  |  |  | + | + | + |  |
| KU.959 | + |  |  |  | + |  |  |  |
| KU.972 | + |  |  |  | + | + | + |  |
| KU.987 | + |  |  |  | + | + | + |  |
| KU.1003 | + |  |  |  | + |  |  |  |
| KU.1031 | + |  |  |  | + |  |  | + |
| KU.1058 | + |  |  |  | + |  | + | + |
| KU.1083 | + |  |  |  | + |  |  | + |

* '+' indicates target products of each markers. Blank cells indicate no target amplicons.
